# Supplementary material for: Mechanisms governing the pioneering and redistribution capabilities of the non-classical pioneer PU.1
Source: Nat Commun. 2020 Jan 21;11:402. doi: 10.1038/s41467-019-13960-2 (PMC6972792; doi:10.1038/s41467-019-13960-2)
Supplement: Supplementary file 7 — Source data [file 41467_2019_13960_MOESM7_ESM.zip › Source_Data/Figure6/Figure6B_MotifScanOutput/ETS1_mutPU.1/homerResults/motif47.similar.html]

motif47

## Information for motif47

G
T
A
C
A
G
T
C
C
G
A
T
A
C
T
G
A
G
T
C
A
C
G
T
A
C
T
G
C
G
A
T
A
C
T
G
  
Reverse Opposite:  

A
G
T
C
C
G
T
A
A
G
T
C
G
T
C
A
A
C
T
G
G
T
A
C
C
G
T
A
A
C
T
G
A
C
T
G
  

|  |  |
| --- | --- |
| p-value: | 1e-28 |
| log p-value: | -6.601e+01 |
| Information Content per bp: | 1.898 |
| Number of Target Sequences with motif | 664.0 |
| Percentage of Target Sequences with motif | 4.92% |
| Number of Background Sequences with motif | 980.9 |
| Percentage of Background Sequences with motif | 2.80% |
| Average Position of motif in Targets | 95.7 +/- 52.2bp |
| Average Position of motif in Background | 102.6 +/- 63.0bp |
| Strand Bias (log2 ratio + to - strand density) | 0.1 |
| Multiplicity (# of sites on avg that occur together) | 1.03 |
| Motif File: | file (matrix) reverse opposite |

### Similar de novo motifs found

|  |  |  |  |  |  |  |  |
| --- | --- | --- | --- | --- | --- | --- | --- |
| Rank | Match Score | Redundant Motif | P-value | log P-value | % of Targets | % of Background | Motif file |
| 1 | 0.761 | G T C A T G A C C T G A A T C G T A G C G C T A C A T G C T A G C T A G C T A G A T C G A T G C | 1e-24 | -55.555021 | 3.40% | 1.80% | motif file (matrix) |
